# Haemorrhagic Presentation of a Craniopharyngioma in a Pregnant Woman

**DOI:** 10.1155/2014/435208

**Published:** 2014-08-05

**Authors:** Cesare Zoia, Andrea Cattalani, Elena Turpini, Viola Marta Custodi, Marco Benazzo, Fabio Pagella, Paolo Carena, Elisabetta Lovati, Pietro Lucotti, Paolo Gaetani

**Affiliations:** ^1^Department of Neurosurgery, IRCCS Fondazione Policlinico San Matteo, Viale Golgi 19, 27100 Pavia, Italy; ^2^Neurosurgery, Department of Clinical Surgical Diagnostic and Pediatric Science, University of Pavia, Viale Golgi 19, 27100 Pavia, Italy; ^3^Department of Otorhinolaryngology, IRCCS Fondazione Policlinico San Matteo, Viale Golgi 19, 27100 Pavia, Italy; ^4^First Department of Medicine, IRCCS Fondazione Policlinico San Matteo, Viale Golgi 19, 27100 Pavia, Italy

## Abstract

*Objective.* Craniopharyngioma is a rare tumour, and, consequently, acute clinical presentation and diagnosis, during pregnancy, of this pathology are quite difficult to find. Only few cases are reported in the literature, and no one describes these two conditions in association. *Methods.* We report a particular case of craniopharyngioma presenting both of the above conditions. *Results.* The patient was successfully operated with endoscopic technique. *Conclusions.* Rare and difficult cases, created by the superposition of different clinical conditions, need multidisciplinary management, with collaboration, integration, and cooperation between different medical specialists.

## 1. Introduction

Craniopharyngioma is a rare tumour, with incidence of 0,13 cases per 100,000 people/year [1], and it is the mostly benign epithelial tumour of the sellar and suprasellar region. In 1857 Zenker first described pathological findings of cells clusters similar to squamous epithelium in the hypothalamic-pituitary region [2]. The term “craniopharyngioma” was coined in 1931 by Charles Frazier and further popularized by Harvey Cushing who described craniopharyngiomas as “the most formidable of intracranial tumours” [3]. Two principal patterns of craniopharyngioma are recognized: papillary and adamantinomatous. This latter pattern is made up by nests and cords of stratified squamous epithelium which are replaced by a layer of columnar cells on the outskirts, and it is characterized by the presence of dystrophic calcifications and cysts containing “motor-oil-like” fluid (brown-yellow cholesterol-rich fluid). The papillary pattern is made up by papillary squamous epithelium, and it is generally without calcifications or cysts [4, 5].

Acute clinical presentation of craniopharyngioma—consisting of sudden headache, nausea, vomiting, cranial nerves palsy, decrease in visual acuity, diabetes insipidus, fever, or reduced level of consciousness—is very unusual, about 13% of all cases [6]. Within the different types of acute presentation, intratumoral haemorrhage is one of the most uncommon; in Table 1 we report the few cases of craniopharyngiomas with intratumoral haemorrhage reported in the international literature [7–18].

Even more rarely craniopharyngiomas manifest themselves with acute clinical presentation during pregnancy; from 1935 to 2013, 8 cases of craniopharyngioma during pregnancy were described in the international literature (Table 2) [19–24].

This report describes a particular case of craniopharyngioma that presents both of the above peculiarities; despite the difficulties caused by intratumoral haemorrhage and pregnancy, this tumour was treated with endoscopic mini-invasive surgery, resulting in a good and early resolution of the mother's new symptoms without negative consequences for the newborn.

## 2. Case Report

**Figure 1:**
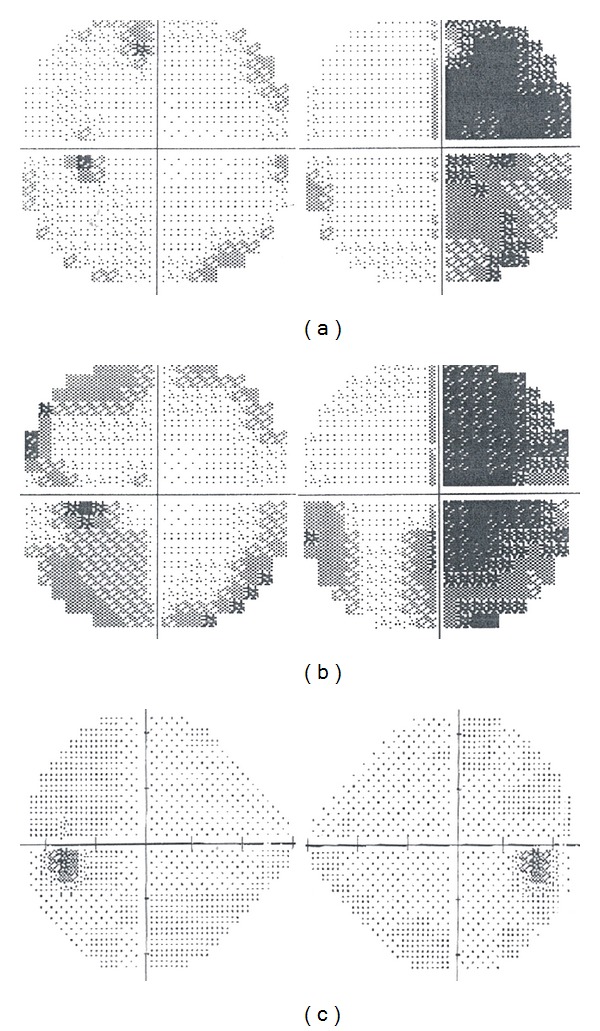
Onset (a), preoperative (b), and postoperative (c) visual field.

**Figure 2:**
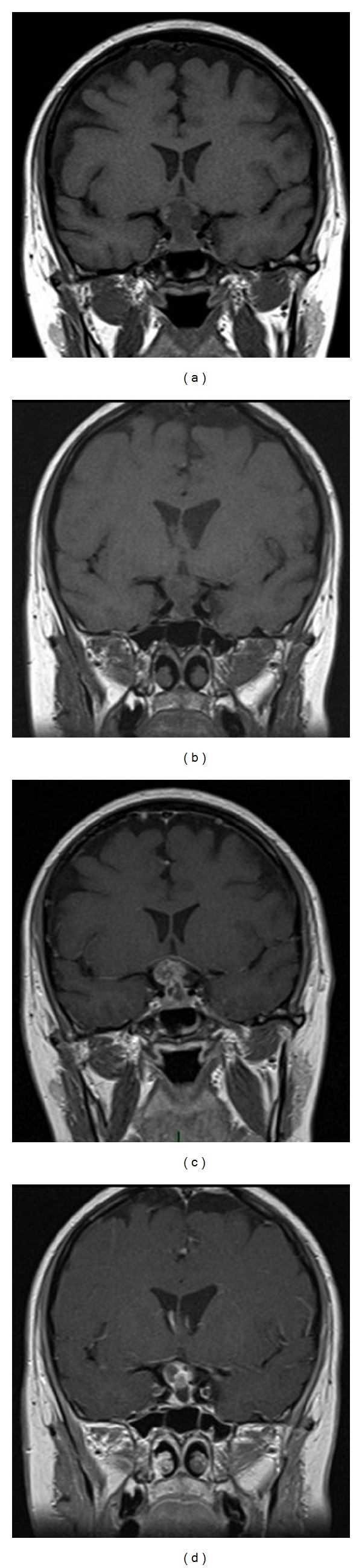
Coronal preoperative ((a) without gadolinium; (c) with gadolinium) and postoperative ((b) without gadolinium; (d) with gadolinium) T1 MRI.

The patient, a 32-year-old woman, became pregnant for the first time in her life. At the 30 weeks + 1 mark of a till-then-normal pregnancy, she presented a sharp bilateral decline in visual acuity. A careful study of her clinical history only noted obesity and Hashimoto's thyroiditis treated with chronic hormone replacement. Then she was assessed by the ophthalmologist: a visual field examination (Figure 1(a)) documented temporal hemianopia in the right eye and inferior-temporal field cut in the left eye. Subsequently she underwent magnetic resonance imaging (MRI) scan, which showed an extra-axial lesion in the intra- and suprasellar regions, isointense in T1-weighted sequence (Figure 2(a)), and isohyperintense in long-TR sequences; these neuroradiological findings could be indicative of pituitary apoplexy, macroadenoma, or craniopharyngioma with signs of intratumoral haemorrhage. This clinical and radiological picture made early surgery mandatory. Neurosurgeons and obstetricians jointly evaluated the case and decided, in agreement with the patient, to postpone the operation until a more advanced gestational age to safeguard the fetal well-being. The patient underwent RDS (respiratory distress syndrome) prophylaxis and maternal-fetal welfare monitoring; endocrinological examination of hormone levels confirmed diagnosis of central hypocortisolism. As a consequence the patient underwent adequate hormone replacement: Cortone Acetate 37.5 mg/day. At 33 weeks + 2 of gestation, the patient complained of a further decline in visual acuity; thus, she underwent a new visual field examination (Figure 1(b)) that turned out to be worse than the previous one. For this reason, after a new collegial evaluation, she underwent delivery via caesarean section (33 weeks + 3). The same day a gadolinium enhanced MRI scan was performed (Figure 2(c)), which confirmed the known sellar lesion with signs of recent intratumoral haemorrhage. The next day the patient underwent endoscopic transsphenoidal subtotal resection of the lesion and decompression of the optic chiasm. The intraoperative appearance of the lesion was that of a hard-elastic mass with fair haemorrhagic component. Histological examination of the collected material deposed for “adamantinomatous craniopharyngioma.” On the VIth postoperative day, nasal liquorrhea appeared and was resolved by positioning a lumbar drainage for 6 days. Transient diabetes insipidus was treated with DDAVP (desmopressin acetate). The patient was discharged on the XVth postoperative day. Currently she is in good clinical condition and an endocrinological examination, performed one month after surgery, has excluded more hormone defects than prior to pregnancy. A visual field examination performed 2 months after surgery confirmed the resolution of the preoperative visual field defect (Figure 1(c)). An MRI scan 3 months after surgery showed a satisfactory decompression of the optic chiasm and a reduction of the tumoral mass (Figures 2(b) and 2(d)).

## 3. Discussion

The international literature counts only few cases of craniopharyngiomas in pregnancy and equally few of intralesional haemorrhage cases in this kind of tumours.

As showed in Table 1, only 14 cases of craniopharyngioma with haemorrhagic presentation are reported in the literature. A considerable amount of the information contained in these articles appears to be incomplete and for this reason it is not possible to determine the average age of the patients or a gender division. In 11 cases (78.5%) the haemorrhage was intralesional, in one case (7.1%) it was subarachnoidal, in one case it was subdural, and in one case the type of haemorrhage was not reported. The clinical presentation was characterized by headache, nausea, and vomit in 6 cases (42.6%), visual disturbances like diplopia, bitemporal hemianopsia, and other visual symptoms in 8 cases (56.8%,), hypopituitarism and diabetes insipidus in 4 cases (28.4%), and neck rigidity or fever in only one case (7.1%). Surgical treatment was transcranial in 4 cases and transnasal with endoscopic technique in 3 cases (21.3%); in the remaining cases there is no information given about the treatment. In 3 cases (21.3%) the visual deficit had been recovered at follow-up; in one case (7.1%) the visual deficit was not recovered; in the other cases no data are given about follow-up.

As shown in Table 2, only 8 cases of craniopharyngioma presenting during pregnancy are reported in the literature. The average age of patients at diagnosis is 27.1 years and in six cases (75%) the presentation was after the 20th week of pregnancy; only in two cases (25%) the presentation was in the first 10 weeks of pregnancy. Symptoms of presentation were visual disturbances in 6 cases (75%), headache in 6 cases (75%), diabetes insipidus in three cases (37.75%), and severe fatigue in 1 case (12.25%). In 2 cases (25%) therapeutic abortion was necessary and in the other 6 cases the delivery was between the 33rd and the 40th weeks (1 caesarian section). In all cases the tumour was resected: in two cases (25%) with transnasal endoscopic technique and in 1 case (12.25%) with a frontotemporal approach, and in the other cases neither the surgical technique nor the entity of the removal is reported. At follow-up, in six cases the patient had recovered the visual deficit after delivery and operation, in one case (12.25%) the patient became blind, and in one case an inferotemporal quadrantopsy was reported.

The case we report is of particular relevance because it is the first to include both of these rarely associated clinical features. Such combination requires a multidisciplinary assessment to allow choosing the most suitable treatment from different specialistic points of view: neurosurgical, neuroradiological, endocrinological, obstetrical, gynaecological, otolaryngological, and ophthalmological.

The role of the first specialist observing the patient is crucial: his task is to correctly identify the location of the problem, in order to set an appropriate diagnostic and therapeutic plan. In the case we report, the first specialist to assess the patient was the ophthalmologist. Bitemporal hemianopia or temporal field cuts in the examination of the visual field are often suggestive of expansive lesions near the optic chiasm and sellar region. A suspicion of such nature needs neuroradiological evidence, obtainable by MRI. In this case, since the patient was pregnant, MRI was performed without contrast: it showed the presence of an extra-axial lesion in the sellar and suprasellar regions. As described by Jagannathan et al., there are many diseases that can develop in these anatomical sites: pituitary adenomas, meningiomas, metastases, abscesses, aneurysms, pituitary apoplexy, and Rathke's cleft cyst in the sellar region and aneurysms, teratomas, hypothalamic gliomas, meningiomas, and epidermoid and dermoid cysts in the suprasellar regions [26]. In the case we report, neuroradiologist formulated three diagnostic hypotheses: pituitary apoplexy, macroadenoma, or craniopharyngioma with signs of intratumoral haemorrhage. At this point, the patient was assessed by a neurosurgeon and an endocrinologist. The neurosurgeon pointed out that the patient needed a surgical intervention as soon as possible, in order to obtain adequate decompression of the optic chiasm and to treat visual impairment. The endocrinologist stressed the importance of a comprehensive study of the hormonal status, to discover and correct any subclinical deficiency. Both of them requested close collaboration with obstetrics. At first, hormonal changes during pregnancy affected the endocrinological treatment. Furthermore, intervention of the neurosurgeon was subject to the timing of delivery; despite the visual disturbances requiring treatment as soon as possible, in this case the most important elements to keep in mind were maternal well-being and fetal growth. So optic chiasm decompression was done a few hours after delivery by caesarean section. In the end, the endocrinologist also had the task of avoiding acute adrenal insufficiency in a patient subjected to two surgical stresses in a few hours. The surgery, in cooperation with ENT surgeons, was performed using the endoscopic endonasal transsphenoidal approach with the “two nostrils four hands technique” [27]. Sellar floor reconstruction was not needed.

The choice of endoscopic treatment was based on the great benefits reported by the international literature during the last years [28–38]: minimal invasiveness, reduced postoperative recovery period, and minimal psychological impact on patient, surgical outcomes, and complication rate similar to those of the classic microscopic technique.

In this case, a patient with a delicate endocrinological balance underwent 2 surgical procedures in few hours: use of endoscopic minimally invasive technique reduced the consequences of this particular surgical stress. Reduced recovery days and early ability in taking care of the newborn also resulted in the patient's more positive psychological reaction to her own disease.

In our opinion, this case represents another excellent demonstration of the versatility of the endoscopic technique, which can be used in complex clinical conditions and considered a gold standard in the surgery of the sellar and paraphrase regions.

The role of the various specialists remains important during follow-up of the patient: the ENT surgeon endoscopically checks for good repair of the wound; the neuroradiologist carries out the necessary radiological postoperative scans (CT in the early postoperative period and MRI 3 months later); the endocrinologist checks the hormonal balance and sets adequate hormone replacement; the ophthalmologist carries out field examinations in the postoperative period. Therefore, the collaboration remains critical among these different medical figures during follow-up.

## 4. Conclusion

We report what, to the best of our knowledge, is the first case in the literature in which two rare features of craniopharyngiomas overlapped at the onset; such synergistic coexistence created a clinical condition difficult to diagnose and manage.

In this particular situation, once diagnosis of sellar lesion had been obtained, all medical and surgical treatments undertaken were found to be necessary and, in particular, endoscopic minimally invasive surgery was considered the first choice. In our opinion this technique represents the gold standard in surgical approach to sellar and parasellar regions and is indispensable in cases like the reported one.

Rare and difficult cases, created by the overlapping of different clinical conditions, need multidisciplinary management, with collaboration and cooperation between many medical specialists: careful preoperative planning, minimally invasive surgery with less possible complications, and adequate follow-up are obtained only with close support and collaboration among all specialists involved.

## Figures and Tables

**Table 1:** Case reports of haemorrhages in craniopharyngiomas.

Author, year	Age, sex of patient	Previous clinical history	Symptoms of presentation	Type of haemorrhage	Hospital course	Histology	Follow-up
Gass, 1956 [7]	<1 yr			Bilateral SDH			

Lloyd and Belchetz, 1977 [8]	29 yrs, F	Secondary amenorrhea and frequent HAs for a few months	HA, vomiting, fever, drowsiness, neck rigidity, right temporal field defect, and hypopituitarism	Intratumoral	1st craniotomy: partial resection, irradiation of the pituitary fossa, hormone replacement, and 2nd reintervention		30 mths later: persistent right temporal field defect, hormone replacement-dependent

Kubota et al., 1980 [9]	>29 yrs			SAH			

Wakai et al., 1982 [10]	>15 yrs						

Kellen et al., 1988 [11]	37 yrs, M	Minor head injury in a car accident	Diplopia	“Secondary” intratumoral		Sq	

Yamamoto et al., 1989 [12]	59 yrs, F	Sciatic pain and mild HA for two months previously	HA, nausea	Intratumoral	Right F-T craniotomy: subtotal resection	Ad	Transient DI

Masuda et al., 1990 [13]	63 yrs, F		Bitemporal hemianopsia, late visual symptoms	Intratumoral	1st transsphenoidal removal of hematoma; other 3 operations: partial resection	Sq	Recurrence, enlargement and histological modification of the tumor

Yousem et al., 1990 [14]	1–23 yrs			Intratumoral			

Yousem et al., 1990 [14]	16 mths		Visual disturbances	Intratumoral			

Makwane et al., 1996 [15]	46 yrs, M		HA, vomiting	Intratumoral			

Ishii et al., 1999 [16]	44 yrs, F		HA, bitemporal hemianopsia, hypopituitarism, and DI	Intratumoral	Transsphenoidal partial resection	Sq	

Nishioka et al., 2000 [17]	49 yrs, F	22 yrs previously right F-T craniotomy, partial resection, and **residual right visual defect**	HA, nausea, anorexia, hypopituitarism, and left visual disturbance	Intratumoral	Hormone replacement, transsphenoidal complete resection	Sq	Left visual function improved immediately after surgery

Yamashita et al., 2004 [18]	22 yrs, M		Declining vision and bitemporal hemianopsia after two lumbar taps, mild DI	Intratumoral	DDAVP, craniotomy with total resection of the tumour and haemorrhage	Sq	Vision returned to normal, hormone replacement

Yamashita et al., 2004 [18]	29 yrs, F	1st transsphenoidal suprasellar resection (vision normalized after surgery)	HA and bitemporal hemianopsia a few months after 1st operation	Intraresidual of the lesion	Craniotomy with partial resection of the haemorrhagic tumour	Sq	Visual field returned to normal, Gamma-Knife for residual

Zoia, 2014	32 yrs, F		Visual problems (temporal hemianopia RE and inferior-temporal field cut LE) during pregnancy	Intratumoral	Delivery via CS, endoscopic transsphenoidal subtotal resection and decompression of the optic chiasm, lumbar drainage for 6 days, and DDAVP	Ad	Vision returned to normal, RMN (3 mths later) small residual, and no chiasm compression

SDH: subdural hematoma; HA: headache; SAH: subarachnoid haemorrhage; Sq: squamous papillary, Ad: adamantinomatous, F-T: frontotemporal; DI: diabetes insipidus; DDAVP: desmopressin acetate, RE: right eye; LE: left eye; CS: caesarean section.

**Table 2:** Case reports of craniopharyngiomas during pregnancy.

Authors, year	Age of patient	Symptoms of presentation	Hospital course	Follow-up
Fischer, 1935 [19]	**?**	Bitemporal HA at 20 wk gestation	Therapeutic abortion	Patient became blind

Sachs et al., 1978 [20]	24 yrs	Visual problems and HA at 28 wk gestation	Tumour resected, DDAVP treatment, and normal term delivery	Vision returned to normal

Van der Wildt et al., 1980 [21]	24 yrs	DI at 20 wk gestation	DDAVP treatment, delivery at 36 wk, and tumour resected postpartum	Vision returned to normal

Hiett and Barton, 1990 [22]	22 yrs	DI at 27 wk gestation, HA, and visual problems	DDAVP treatment, delivery at 34 wk, and tumour resected postpartum	Vision improved after tumour resection

Johnson et al., 1993 [19]	27 yrs	Visual problems, HA in 2nd trimester	Tumour resection, normal term delivery	Vision near normal after tumor resection

Maniker and Krieger, 1996 [23]	35 yrs	Visual problems, HA at 8 wk gestation	2 transsphenoidal resections, healthy delivery via CS at 33 wk	Vision returned to normal

Aydin et al., 1999 [25]	19 yrs	Visual problems and HA at 20 wk gestation	Transsphenoidal resection, delivery at term	Vision returned to normal, 2nd resection during subsequent pregnancy 4 yrs later

Magge et al., 2001 [24]	39 yrs	Visual problems, DI, and severe fatigue at 6 wk	Abortion, F-T craniotomy with suprasellar resection, and intranasal DDAVP	Small inferior temporal quadrantanopsia in LE, 2nd pregnancy with new bitemporal field cut that disappeared after normal vaginal delivery, DI in treatment with DDAVP, and thyroid hormone replacement

Zoia, 2014	32 yrs	Visual problems (temporal hemianopsia RE and inferior-temporal field cut LE) at 30 wk + 1 gestation	Cortisol replacement, delivery via CS at 33 wk + 3, endoscopic transsphenoidal subtotal resection and decompression of the optic chiasm, lumbar drainage for 6 days, and DDAVP	Vision returned to normal, no hormone deficiencies; RMN (3 months later) shows small residual with no chiasm compression

HA: headache; DI: diabetes insipidus; CS: caesarean section; F-T: frontotemporal; DDAVP: desmopressin acetate; RE: right eye; LE: left eye.
